# Exploring the Prognostic Significance and Immunotherapeutic Potential of Single-Cell Sequencing-Identified Long Noncoding RNA (LncRNA) in Patients With Non-small Cell Lung Cancer

**DOI:** 10.7759/cureus.48436

**Published:** 2023-11-07

**Authors:** Ling Chen, Lina Wang, Zhuolong Xiong, Xiao Zhu, Lianzhou Chen

**Affiliations:** 1 Laboratory of General Surgery, The First Affiliated Hospital, Sun Yat-sen University, Guangzhou, CHN; 2 Department of Genetics, The Marine Biomedical Research Institute, Guangdong Medical University, Zhanjiang, CHN; 3 Department of Clinical Laboratory, Qingdao Sixth People’s Hospital, Qingdao, CHN; 4 Laboratory of Computational Oncology, The Marine Biomedical Research Institute, Guangdong Medical University, Zhanjiang, CHN

**Keywords:** single cell sequencing, response to medication, prognosis, non-small cell lung cancer, immune system markets

## Abstract

Background: Single-cell RNA sequencing technology can provide insight into lung cancer. The purpose of this study was to analyze the relationship between long noncoding RNA (lncRNA) discovered by RNA sequencing and immunotherapy in patients with non-small cell lung cancer (NSCLC).

Methods: In this study, we utilized data from The Cancer Genome Atlas (TCGA) to extract gene expression data and prognostic information from patients with NSCLC. We employed univariate, least absolute shrinkage and selection operator (LASSO), multivariate Cox regression analyses to construct risk models, and Kaplan-Meier (KM) analysis to compare survival differences between high- and low-risk groups. To evaluate the accuracy of our risk model predictions, we utilized a nomogram, calibration curve, correlation index curve (C-index), and receiver operating characteristic (ROC). Additionally, we conducted Gene Ontology (GO) enrichment analysis and Kyoto Encyclopedia of Genes and Genomes (KEGG) enrichment analysis to investigate the differential expression of lncRNA genes. We also used the tumor immune dysfunction and exclusivity (TIDE) algorithm and the R package "pRRophetic" to analyze the tumor microenvironment. Finally, we utilized stem cell indices based on mRNA expression-based stemness index (mRNAsi) expression to better assess patient prognosis.

Results: Our analysis identified a set of 28 lncRNAs with prognostic risk profiles in patients with lung adenocarcinoma. Notably, patients in the low-risk group exhibited significantly better overall survival (OS) compared to those in the high-risk group. Kaplan-Meier (KM) survival curves revealed that these prognostic risk markers accurately predicted survival outcomes in non-small cell lung cancer (NSCLC) patients. MerCK18 and myeloid-derived suppressor cells (MDSC) were strongly associated with immune escape and immunotherapy in high- and low-risk subgroups. In our investigation of potential chemotherapeutic agents for the treatment of NSCLC, we screened a total of 60 agents and found that PPM1D was more effective in the low-risk group. However, we did not observe a strong correlation between the stem cell index mRNAsi and OS.

Conclusion: Our study highlights the close association between lncRNAs and prognostic risk profiles and the prognosis of patients with non-small cell lung cancer, offering a promising avenue for the clinical implementation of immunotherapy.

## Introduction

Single-cell sequencing is a technique that can reveal the dynamic changes in the genome, transcriptome, or epigenetics of a specific cell [1]. In contrast to traditional sequencing methods that sequence a large number of mixed cells and average the information, single-cell sequencing technology is not only more accurate but also more comprehensive and multi-level. Therefore, it is very helpful to use single-cell sequencing technology to study tumors and understand their occurrence, development, and evolution. In 2009, single-cell sequencing technology was first cited, and it solved the problem of sequencing sample heterogeneity to a certain extent [2]. In 2013, researchers used single-cell sequencing techniques to sequence single circulating tumor cells in the peripheral blood of tumor patients, providing a new means of tumor diagnosis and monitoring [3].

As the second leading cause of death in the world, tumors are local masses formed by the abnormal proliferation of local tissue cells under the influence of various carcinogenic factors [4]. Lung cancer is becoming an increasingly serious problem in developing countries due to the growing number of smokers and the expanding scope and extent of air pollution [5]. In China, lung cancer ranks first in the incidence and mortality of malignant tumors, and the survival rate of lung cancer is closely related to the stage at the time of detection [6]. Non-small cell lung cancer (NSCLC) accounts for approximately 85% of new cases of lung cancer, and its five-year survival rate is less than 16%. [7]. For non-small cell lung cancer, lung adenocarcinoma (LUAD) represents the most prevalent histological cell subtype of NSCLC. As the tumor grows, changes occurring after multiple divisions and proliferation lead to differences in tumor growth rate, invasion ability, sensitivity to drugs, prognosis, and other aspects, resulting in tumor heterogeneity [8]. In a study by Kim et al., 83 cells were extracted from tumor cell implantation mice (patient-derived xenograft (PDX)) in different lung adenocarcinoma patients for sequencing. They performed a full exon strategy on duplicate samples and found that all PDX cells were heterogeneous [9]. Tumor heterogeneity is a key problem in tumor research and diagnosis, and single-cell sequencing technology can identify genetic variations and their variation rate in the process of tumor development, distinguish immune cell subsets in tumors, and explore the process of tumor occurrence [10].

Different types of tumor-associated gene mutations have been clinically applied as markers for the diagnosis of specific types of tumors, including mutations in the BRAF gene in melanoma and estimated glomerular filtration rate (eGFR) gene mutations in non-small cell lung cancer [11]. Single-cell sequencing technology can detect the heterogeneity of tumor cells, search for genes based on specific marker genes found under the immune system, draw the relevant genetic map, and interpret the expression of tumor cells under the immune system, which can be valuable for clinical exploration of diagnosis and treatment.

## Materials and methods

Gene source

Genes are the fundamental building blocks of genetic variation in the human body, supporting the basic structure and function of life. It has been argued that genes are the order of monomers in the final functional polypeptide or RNA molecule or a group of closely related subtypes [12]. Therefore, the collection of genes is an indispensable part of our research. We utilized single-cell RNA sequencing technology and Panglao DB, the most user-friendly and widely used online database, which contains data from more than 4 million cells [13]. We then subtracted the repeatedly calculated genes, noncoding genes, and pseudogenes by single-cell sequencing technology, resulting in a total of 3,066 genes, with 2,579 genes being immune cell markers (http://biocc.hrbmu.edu.cn/CellMarker/index.jsp).

Sources of data

We obtained gene expression, clinicopathological, and prognostic data for 494 NSCLC patients from The Cancer Genome Atlas (TCGA) (https://portal.gdc.cancer.gov/). The Cancer Genome Atlas results indicate that a detailed analysis of clinical tumor specimens is required to obtain a specific set of mutations, which is beneficial for patient selection for targeted therapy [14]. Comprehensive genomic data from a large number of patients contribute to more effective diagnosis, treatment, and prevention [15]. Finally, we used the "limma" package with R software (version 4.0.2, The R Core Team, R Foundation for Statistical Computing, Vienna, Austria) to obtain all the gene sets and visualize them with a Sankey diagram.

Construction of long noncoding RNAs (lncRNAs)-related prognostic model

Initially, we conducted a univariate Cox regression analysis to identify significant genes in the entire cohort of NSCLC patients. Subsequently, we randomly divided the patients into a training cohort (comprising 330 patients) and a test cohort (comprising 164 patients). In the training cohort, we performed univariate Cox regression analysis to establish the correlation between lncRNAs and overall survival (OS) in NSCLC patients. Genes that were significantly associated with OS (P<0.05) were selected as prognostic genes. To account for multicollinearity among variables, we employed the least absolute shrinkage and selection operator (LASSO) Cox regression analysis. Finally, we constructed prognostic signatures based on the potential candidate lncRNAs obtained from the above screening using multivariate Cox regression analysis. The patient's risk score was estimated using the following formula:

 (Risk score= ∑_(i=1)^n〖Coe f_i 〗* x_i)

Here, the coefficients and the expression levels of the selected lncRNAs were estimated through multivariate Cox regression analysis. We plotted the receiver operating characteristic (ROC) curve and the risk score graph to evaluate the predictive performance of the model. Using the median risk score as the cut-off value, we divided the NSCLC patients in the training cohort into high-risk and low-risk subgroups. The Kaplan-Meier (KM) method and log-rank test were employed to analyze the OS of the high-risk and low-risk subgroups.

Construction of lncRNAs prognostic risk scoring system

To construct the lncRNAs prognostic risk scoring system, we initially extracted the expression levels of the gene set and the co-expressed lncRNAs and visualized their co-expression relationship using a Sankey diagram. We then performed survival analysis using a univariate Cox proportional hazards model in R to identify lncRNAs with overlapping differential expressions, with a screening condition of a p-value less than 0.05. A multivariate Cox proportional hazards regression analysis was subsequently conducted to further identify candidate genes with the same screening conditions. The data were randomly divided into a training group and an experimental group, with good grouping achieved if the statistical p-value between the clinical information of the two groups was greater than 0.05 and there was no statistical offset error.

Clinical independent prognostic model analysis and visualization

To validate the prognostic risk model's ability to predict patients' survival, we evaluated the quality of the clinical independent prognostic model using both the clinical ROC curve and the receiver operating characteristic (ROC) curve of survival time [16], which were highly accurate. Most of the clinical indicators, except for age and gender, were found to predict the five-year survival probability of patients. The accuracy of the model was considered high when the area under the ROC curve (AUC) was greater than 0.05, with higher values indicating greater accuracy. The clinical concordance (C)-index curve was also used to evaluate the quality of the clinical independent prognostic model, with a higher index indicating better predictive ability. We further used a nomogram, a concise and convenient graph, to predict the patient's survival probability and evaluated the predictive quality of this model's nomogram using the calibration curve method.

Validation of prognostic risk model in a clinical grouping of patients

To construct the lncRNAs prognostic risk scoring system, we began by extracting the expression levels of the gene set and the co-expressed lncRNAs. We then visualized their co-expression relationship and performed prognostic risk modeling in subgroups with different clinical characteristics, including age, gender, race, survival status, the American Joint Committee on Cancer (AJCC) stage, primary tumor (T), regional lymph nodes (N), and distant metastasis (M). This verified that there was no difference between the high- and low-risk subgroups among the variable groups. Next, we used principal component analysis (PCA) to verify whether there was discriminative attention between coding genes and non-coding genes between high- and low-risk subgroups. Using mRNA as a distinction, the blue distinction between patients in the low-risk group and red in the high-risk group was obvious, and we can prove that there is no distinction.

Differential expression analysis of lncRNAs in patients with high and low tumor risk models

To further study the genes, we showed the expression differences and p-values of non-coding genes between the high and low-risk groups using R packets. All the differential expressions were included in the Gene Ontology (GO) and Kyoto Encyclopedia of Genes and Genomes (KEGG) pathway analyses, which were performed using the R package. Gene ontology and KEGG are all in the annotations, visualization, and integration found in the database (Database for Annotation, Visualization, and Integrated Discovery (DAVID)) (https://david.ncifcrf.gov/).

Analysis of tumor microenvironment in low-risk and high-risk groups

We then performed immune function studies on samples in the training group through R software, followed by studying the immune function of the validation group and all samples. The Gene Set Variation Analysis (GSVA) package in the R package was used to analyze the gene set, setting the threshold to an adjusted P<0.05. Gene Set Variation Analysis is a gene set variation analysis method that calculates the sample gene set score as a function of the genes inside and outside the gene set, similar to the competitive gene set test [17]. Following the examination of immune function, the tumor mutation burden (TMB) was compared between the high- and low-risk groups, and its impact was visualized through survival curves. Tumor mutation burden typically represents the total number of mutations in tumor specimens, serving as a simple measure to evaluate the frequency of gene mutations [18]. Immunotherapy proves more effective when accompanied by a higher TMB value [19]. Subsequently, an analysis was conducted on immune evasion, immunotherapy, and the definition of tumor immune dysfunction and rejection (TIDE) algorithms. The data utilized in this analysis were sourced from the TIDE web platform. Furthermore, the microsatellite instability (MSI) score, an important clinical marker for tumor diagnosis, was examined, as it can also serve as an indicator for evaluating the efficacy of immunotherapy, chemotherapy, or radiotherapy [20]. Additionally, other immune biomarkers or cells were analyzed, including Merck18, CD274, interferon-gamma (IFNG), CD8, myeloid-derived suppressor cells (MDSC), cancer-associated fibroblast (CAF), and tumor-associated macrophage M2 (TAMM2). Lastly, the "pRRophetic" package of tumor chemotherapy drugs, screened using R software, was employed, maintaining the order of analysis for the training group, validation group, and all samples. The data utilized in this process were obtained from the database (http://tide.dfci.harvard.edu/) [21].

The calculation of the lung cancer stem cell index

The characteristics of stem cells were identified using the single logistic regression (one-class logistic regression (OCLR)) algorithm. By applying these stem cell characteristics to RNA-seq data, a stem cell index was derived [22]. This index serves as a measure of similarity between tumor cells and stem cells [23]. The stem cell index of each patient was determined through differential analysis of the LUAD stem cell index. A higher stem cell index indicates a more malignant tumor [24]. A stem cell index of 0 signifies low similarity with stem cells, while a value of 1 indicates a higher similarity, reflecting stronger stem cell characteristics. The stem cell index is directly correlated with the progression of various cancer types. Previous studies have explored the functional implications of the stem cell index in lung cancer, identifying specific genes and pathways associated with the immune system that contribute to our understanding of the potential relationship between cancer stemness and the lung cancer microenvironment [25]. Finally, survival analysis was performed between the high- and low-stem cell index groups; the relationship between the stem cell index and clinicopathological features was tested, and the results were visualized.

## Results

Relationship between gene set and lncRNA

A univariate Cox regression analysis revealed that 230 lncRNAs were significant (p-value<0.05). Among them, MIR4527HG and FRMD6-AS1 were significantly expressed with a p-value of less than 0.001 (Table 1).

**Table 1:** Results of the univariate Cox regression analysis in the entire cohort The table presents p-values, hazard ratios (HR), and confidence intervals (CI) for the 53 differentially expressed genes in the entire cohort.

Gene	HR	HR.95L	HR.95H	p-value
LINC01312	1.899501027	1.578384814	2.285947076	1.12E-11
LINC00941	1.037225559	1.026203577	1.048365923	2.01E-11
AC080023.1	1.635257455	1.377763234	1.940875527	1.85E-08
LINC01537	1.306531555	1.184129093	1.441586661	9.96E-08
LINC00707	1.021070557	1.012762422	1.029446847	5.67E-07
LINC02448	1.192624712	1.109879266	1.281539124	1.57E-06
AC108136.1	1.073737122	1.04254361	1.105863961	2.25E-06
SIRLNT	1.048769689	1.027936007	1.070025617	3.30E-06
BZW1-AS1	1.527358042	1.272597466	1.833118996	5.39E-06
AC025419.1	1.022107018	1.012496922	1.031808328	5.71E-06
SMILR	1.045403997	1.025164542	1.066043031	8.53E-06
ABCA9-AS1	1.567709086	1.283962628	1.914161458	1.02E-05
AL606489.1	1.08410455	1.045372141	1.124272047	1.36E-05
TMPO-AS1	1.099220753	1.050127106	1.150609539	4.95E-05
LINC00519	1.045403901	1.022542401	1.068776527	8.29E-05
MIR4527HG	1.121364663	1.05771455	1.188845051	0.000122069
FRMD6-AS1	1.584761118	1.250944422	2.007657379	0.00013606
AC083967.1	1.046358692	1.022015394	1.071281822	0.000161214
GSEC	1.047876551	1.02258318	1.073795548	0.000175902
AC027088.3	1.72395926	1.296920877	2.291608983	0.000176642
DIRC3	1.117901687	1.054523193	1.185089329	0.000182015
AL161668.1	1.026161423	1.012255933	1.040257935	0.000207359
AF121898.1	1.138634374	1.059795219	1.223338448	0.000390672
AC133785.1	1.15947705	1.06793581	1.258865015	0.000421292
AC004704.1	1.020940511	1.009200998	1.032816583	0.000444555
AC010343.3	1.121047473	1.051388163	1.195322033	0.000481295
LINC00598	1.282973887	1.11474256	1.476593838	0.000511547
LINC00628	1.882836486	1.315096629	2.695675098	0.00054841
AP000695.1	1.064896756	1.027451617	1.103706572	0.00057579
AC046143.1	1.1011228	1.042017881	1.163580245	0.000621294
AC011611.2	1.386943343	1.149764428	1.673048661	0.000629853
LINC02310	1.197449903	1.079869984	1.327832324	0.000632815
HLA-DQB1-AS1	0.975567788	0.961741079	0.989593281	0.000682884
AC005291.2	1.021318746	1.00887915	1.033911724	0.000741416
AC005034.3	1.037577236	1.015412905	1.060225367	0.00081311
TM4SF19-AS1	1.125328414	1.049665165	1.206445714	0.000884627
AC034223.2	1.045836998	1.018342939	1.074073363	0.000976443
AL162632.3	3.695901635	1.69840779	8.042643809	0.000983583
AC026355.2	0.916802154	0.870389078	0.965690184	0.001048816
IL12A-AS1	1.099924253	1.038743831	1.164708107	0.001107114
AC004080.2	1.082934336	1.032151923	1.13621527	0.00114846
LINC02587	1.01053917	1.00416145	1.016957397	0.001172202
LINC00602	1.748816911	1.236888506	2.472624308	0.001561119
LINC01747	2.277931742	1.366151381	3.798241611	0.001599421
MIR1915HG	1.100013038	1.036556841	1.167353913	0.001664734
LINC01385	1.081698859	1.029928338	1.136071686	0.001698365
LINC01415	1.373092184	1.125922437	1.674522227	0.001740627
AL121820.1	1.158074104	1.056123853	1.269865864	0.001800263
AC087752.3	0.849131091	0.766026139	0.941251966	0.001857739
AC018695.6	1.126603708	1.04465322	1.214983011	0.001976918
LINC00377	1.322247484	1.106745719	1.57971102	0.002088727
AL138789.1	1.038011263	1.013629782	1.062979207	0.002096051
AC018647.1	0.312013376	0.147920145	0.658141232	0.002224433

Construction of prognostic model for lncRNAs

The training cohort (n=330) and test cohort (n=164) were randomly assigned from a total of 494 patients, with no significant differences in clinical characteristics (p-value>0.05). We used univariate Cox regression analysis and LASSO Cox regression analysis to screen lncRNAs and found 53 genes in the training cohort of NSCLC patients. A multivariate Cox regression analysis identified 28 genes (Table 2) that were used to calculate the risk score for each patient in the training cohort.

**Table 2:** Results of multivariate Cox regression analysis The table presents p-values, hazard ratios (HR), and confidence intervals (CI) for the 28 differentially expressed genes, along with specific data for the risk score.

Gene	Coef	HR	HR.95L	HR.95H	p-value
AC105290.1	-0.532621429	0.587064004	0.45287444	0.761014786	5.76E-05
LINC01776	1.847416425	6.343409659	2.366133374	17.00616142	0.000240953
`ID2-AS1`	1.136571236	3.116065773	1.681118143	5.775837911	0.000306457
`MAP3K20-AS1`	0.720059278	2.054554997	1.371133676	3.078617577	0.000483648
AC010999.2	-1.283685662	0.277014435	0.129357159	0.593218015	0.000953128
AC025419.1	0.403863515	1.497599533	1.169766429	1.917309564	0.001355714
AC025741.1	1.807581713	6.095688468	1.931188672	19.24069794	0.002054932
AC022165.1	1.269616343	3.559486679	1.583498927	8.001233977	0.002125018
AL157931.1	0.359971679	1.433288822	1.125038416	1.825997065	0.003573338
AL031600.2	-1.294460022	0.274045803	0.108939088	0.689386186	0.005955535
LINC02410	-1.140831881	0.319553081	0.132753135	0.76920347	0.010914256
`IER3-AS1`	0.448307574	1.565660178	1.10636212	2.21563243	0.011389971
LINC02448	0.502895842	1.653502627	1.116831233	2.448060957	0.012009428
AC026310.2	-2.231169127	0.107402789	0.017423661	0.662051389	0.016199239
`PAN3-AS1`	-0.719199879	0.487141873	0.267503132	0.887119349	0.018692857
AC018529.1	-0.719078295	0.487201105	0.26244465	0.904438009	0.0227157
AP003500.1	-0.464770958	0.628278991	0.4186392	0.942899018	0.024844016
C5orf66	1.007997951	2.740109686	1.117427892	6.719181746	0.027624762
AL139099.3	0.350570459	1.4198773	1.031068053	1.955304058	0.031763782
FAM66C	0.652265399	1.919885213	1.021785784	3.607369851	0.042668841
`ZNF571-AS1`	-0.340301154	0.711556002	0.506595663	0.999439951	0.049623426
AC012409.4	0.60812153	1.836977448	0.955674736	3.530998589	0.06815514
LINC01150	-0.319576262	0.726456799	0.510630326	1.033505952	0.075611837
AC015967.1	-0.801609214	0.448606479	0.185165039	1.086856212	0.075817632
AC092809.3	0.266508432	1.305398596	0.966061936	1.763929858	0.082711461
AC080023.1	0.440634327	1.553692454	0.908225032	2.657887811	0.107714344
AC105036.3	0.507785967	1.661608264	0.854033378	3.232826837	0.134830703

**Figure 1:**
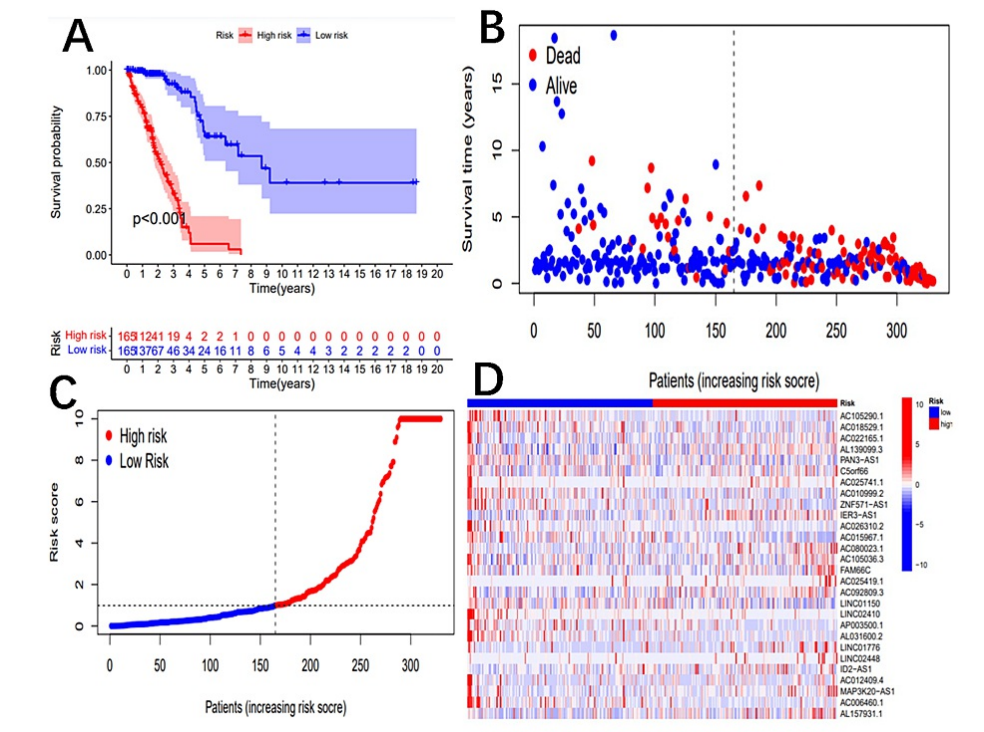
Construction of a 28-lncRNA signature in the training cohort The Kaplan-Meier test revealed a longer survival time for patients in the low-risk group (A). Survival status and risk scores are displayed for each case (B, C). A heatmap illustrates the gene expression, with increasing risk scores depicted from blue to red. AL133445, LINC01754, and AC090236.2 are expressed at lower levels in the high-risk subgroup and at higher levels in the low-risk subgroup (D). lncRNA: long noncoding RNA

Patients were then divided into high-risk (n=165) and low-risk (n=165) subgroups based on the median risk score. The KM survival curve analysis showed that low-risk NSCLC patients had significantly longer survival times than high-risk NSCLC patients (p<0.001) (Figure 1A).

The survival rate of the high-risk subgroup was significantly lower than that of the low-risk subgroup, with higher risk scores indicating a higher risk of death (Figures 1B, 1C). The heatmap displayed the differential expression of 28 risk-related lncRNAs in the high-risk and low-risk subgroups. Genes AC025419.1 and LINC02448 were more highly expressed in the high-risk subgroup, indicating a higher risk of death with increased gene expression. Additionally, a large proportion of positive correlations were observed between the coding and non-coding lncRNAs (Figure 1C). The data were randomly divided into the training and experimental subgroups, with all p-values in the table greater than 0.05, indicating good randomization and no statistical differences between clinical indices that could lead to statistical deviation. On the contrary, LINC02410 exhibits higher expression levels in the low-risk area, indicating that higher gene expression is associated with a lower risk of death, making it a valuable gene (Figure 1D).

Validation of prognostic risk markers

**Figure 2:**
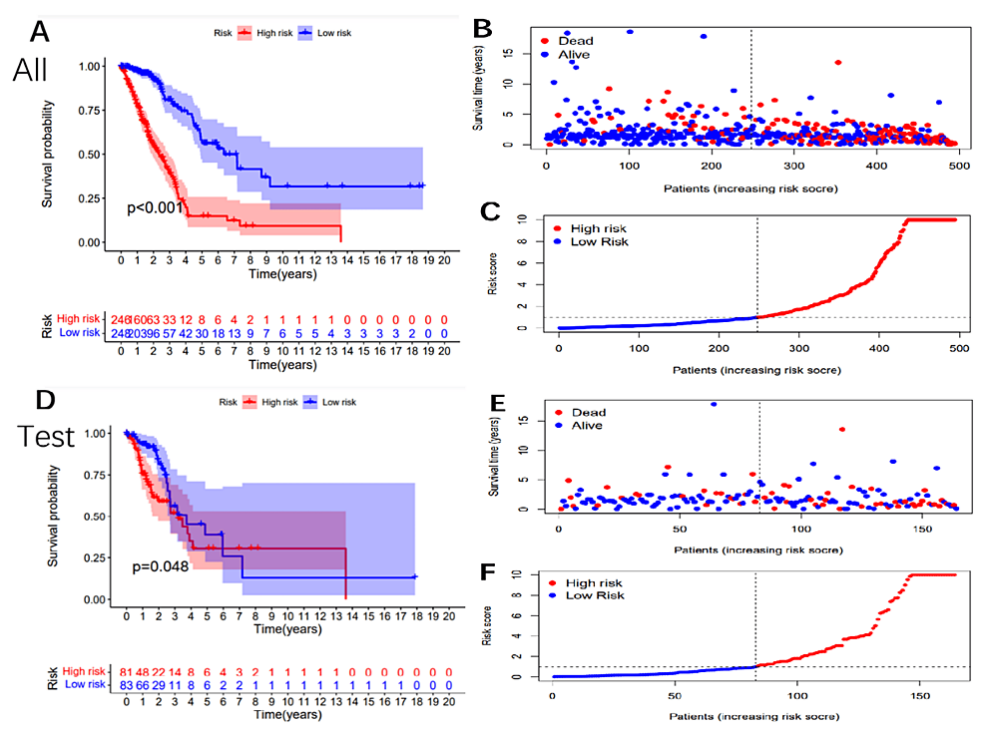
Validation of the 28-lncRNA signature in the testing cohort, training cohort, and entire cohort Overall survival curves (A), distribution of risk scores (B), and survival status (C) are shown for the entire cohort. Overall survival curves (D), distribution of risk scores (E), and survival status (F) are shown for the testing cohort. lncRNA: long noncoding RNA

To ensure the accuracy of our prognostic risk markers, we validated their stability and correctness in both the test group and the entire cohort. Using these markers, we calculated the prognostic risk score for each patient and divided them into high-risk (n=165) and low-risk (n=165) subgroups. The KM survival curve analysis demonstrated that medium-high-risk patients had significantly shorter overall survival than low-risk patients in both groups (p<0.001; Figure 2A, p=0.048 in the test group).

The distribution of the risk score and survival status of each case is shown in Figures 2B-2F. Ultimately, our findings indicate that these prognostic risk markers can accurately predict survival outcomes in NSCLC patients.

Relationship between genes and clinicopathological parameters in patients with non-small cell lung cancer

We also visualized the relationship between genes and clinicopathological parameters in NSCLC patients, revealing that gender (P =0.34), age (P =0.39), race (P >0.05), and M (P =0.1) were not associated with risk scores. Early tumors were associated with the low-risk group, while advanced tumors were significantly associated with the high-risk group.

Prognostic model of patient's clinical information

**Figure 3:**
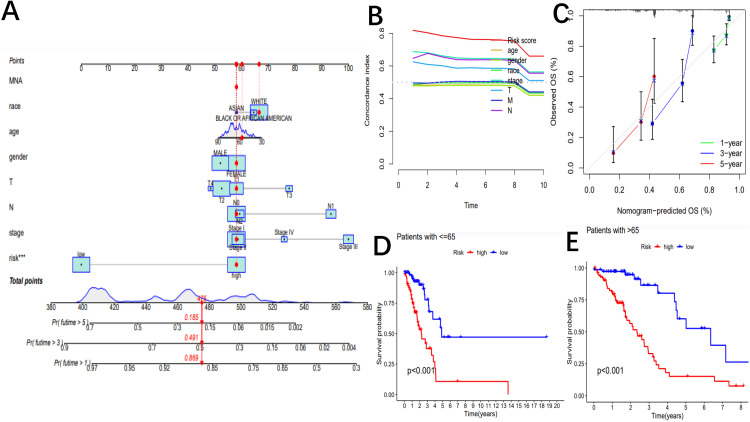
A prognostic model is constructed by integrating the risk score and clinicopathological factors. The C-index curve revealed significant prognostic effects (A). A nomogram was developed to predict the survival rate of NSCLC patients. A vertical line is drawn from the variable value to the axis labeled "point". Points were then calculated across all variables. The total number of points on the bottom scale corresponds to one-year, three-year, and five-year survival rates. "***" indicates that the p-value is <0.001 (B). A calibration curve was plotted to assess the performance of the overall survival model across the cohort. The white diagonal line represents the ideal calibration, while the green, blue, and red lines depict the observed calibration for one year, three years, and five years, respectively (C). The Kaplan-Meier survival curves were used to compare the overall survival of patients with high- and low-risk NSCLC, stratified by clinicopathological parameters. The subgroups were stratified by age ≤65 (D) and >65 (E). C-index: concordance index; NSCLC: non-small cell lung cancer

To further validate the predictive value of lncRNAs in determining patient survival and stability, we constructed multivariate Cox regression models using clinical registry data of tumors as variables, including age, sex, race, survival status, AJCC stage, T stage, N stage, and M stage. The risk score was found to be closely associated with patient survival. In the entire cohort, univariate Cox regression analysis revealed that several factors, including AJCC staging, T staging, N staging, and risk score, were significantly associated with overall survival (OS). Furthermore, the results indicated that clinicopathological factors had significant prognostic value for the one-, three-, and five-year survival rates of NSCLC patients (1-year AUC=0.836, 3-year AUC=0.806, and 5-year AUC=0.799). The risk score (AUC=0.836), gender (AUC=0.554), stage (AUC=0.698), T (AUC=0.633), and N (AUC=0.652) were statistically significant in determining the one-year survival rate of NSCLC patients. In conclusion, the ROC curve demonstrated that the risk score is a reliable predictor of survival at one, three, or five years, although other factors should also be considered as reference factors. The clinical C-index curve showed that the indexes of the clinicopathological stage, N, and T were greater than 0.5, indicating good predictive ability (Figure 3A).

This is consistent with the ROC curve. After considering clinicopathological covariates, we opted to create a nomogram to construct an intuitive prediction model. Based on univariate and multivariate Cox regression analyses, we developed nomograms to predict OS at one, three, and five years (as shown in Figure 3B). The simple and intuitive nomogram clearly indicates that the survival probability decreases from 0.869 in one year to 0.185 in five years if the total clinical characteristics score of the patient is 475, and the probability decreases with time. Meanwhile, the calibration curve verified that our nomogram's prediction quality was good (as shown in Figure 3C).

Model validation of clinical subgroup data

**Figure 4:**
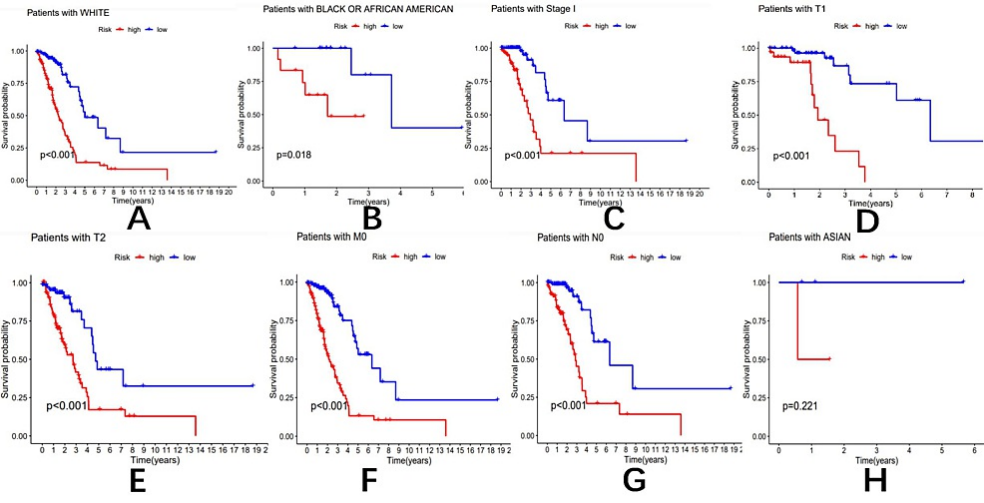
Overall survival of patients with high- and low-risk NSCLC stratified by clinicopathological parameters The Kaplan-Meier survival curves showed the OS rates of high- and low-risk subgroups stratified by race: White (A), race: Black or African American (B), AJCC stages I (C), T1-T2 stage (D, E), M0 stage (F), N0 stage (G), and race: Asian (H). NSCLC: non-small cell lung cancer; OS: overall survival; AJCC: American Joint Committee on Cancer

In terms of model validation of clinical subgroup data, we observed that patients younger than 65 years of age (p <0.001) and patients older than 65 years of age (p <0.001) (as shown in Figure 3D-3E), White patients (p <0.001), Black or African American patients (p=0.018) (as shown in Figure 4A and B), stage I patients (p <0.001) (as shown in Figure 4C), T1 patients (p <0.001) (as shown in Figure 4D), T2 patients (p <0.001) (as shown in Figure 4E), M0 patients (p <0.001) (as shown in Figure 4F), and N0 patients (p <0.001) (as shown in Figure 4G) were differentiated between high and low-risk groups.

Low-risk patients had significantly reduced overall survival. Asian patients (p =0.221) (as shown in Figure 4H), stage II patients (p =0.020), stage III patients (0.006), stage IV patients (p=0.765), stage T3 patients (p=0.009), and stage T4 patients (p=0.422), M1 stage (p=0.765), N1 stage (p=0.090), and N2 stage (p=0.012). These survival analysis results showed no statistical significance, and the OS rate was not associated with a risk score.

**Figure 5:**
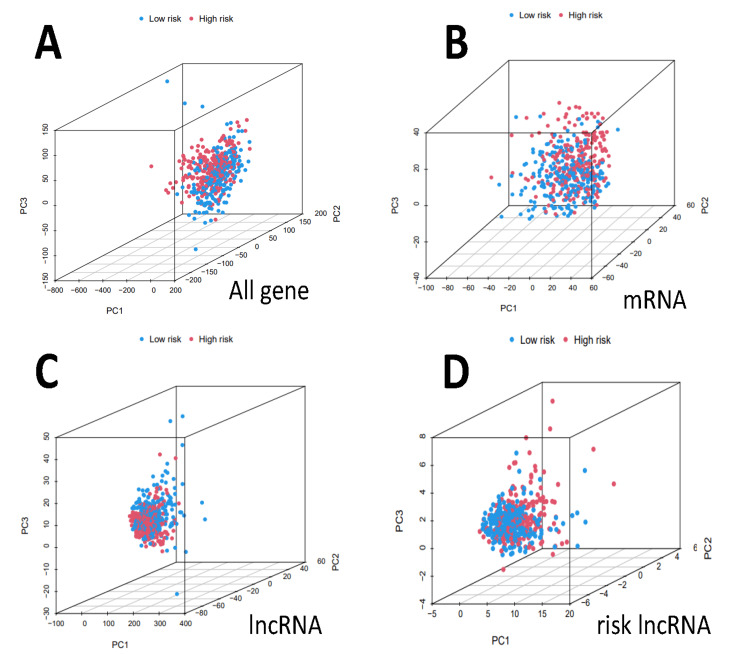
Principal component analysis It revealed gene-expression disparities between high- and low-risk subgroups in all genes (A), mRNA (B), lncRNA (C), and risk lncRNA (D). lncRNA: long noncoding RNA

Using three-dimensional PCA principal component analysis based on coding genes, non-coding genes, and all genes, the low-risk and high-risk populations were segregated into two distinct groups. This was achieved by utilizing lncRNAs to divide patients, indicating significant differences between the low-risk and high-risk subgroups. Furthermore, the two subgroups also exhibited significant differences based on the sum of coding and noncoding genes. Thus, it is evident that there were significant differences in risk-related lncRNAs (Figure 5).

Analysis of lncRNAs gene function in patients with a high and low tumor risk model

To further elucidate the underlying biological functions and major signaling pathways of the genes, functional enrichment analysis, including GO and KEGG analysis, was performed. We compared the expression of lncRNA in the low-risk subgroup and the high-risk subgroup and extracted meaningful lncRNAs. Up to 26 lncRNAs were directly bound up with pathways in GO pathway enrichment results, such as (GO: 0030546: signaling receptor activator activity, 0048018: receptor ligand activity, 0009914: hormone transport). In particular, we need to pay attention to GO: 0030546: signaling receptor activator activity and 0048018: receptor ligand activity, which may be more closely related to lung cancer. In addition, KEGG analysis revealed the potential biological relationships between our gene set and complement and coagulation cascades, nitrogen depletion, and mature-onset youth diabetes mellitus, such as (KEGG: HSA04610: complement and coagulation cascades, MAP05146: amoebiasis, MAP04152: AMP-activated protein kinase (AMPK) signaling pathway).

Immune function of the model

The heatmap displays 13 immune function pathways in the high-risk and low-risk subgroups. Recent research on immune deficiency has identified various immune molecules whose deficiency may cause various diseases. While many of these molecules are necessary for immune function, some are specifically functional [26]. In the training group, we found that human leukocyte antigens (HLA), cytolytic activity, and inflammation-promoting molecules were highly expressed in the low-risk subgroup and lowly expressed in the high-risk subgroup, indicating that they are low-risk pathways. We observed that the expression profile of immune functional pathways was consistent with that of the training cohort in both the test and the entire cohort.

Analysis of differential tumor mutation burden

**Figure 6:**
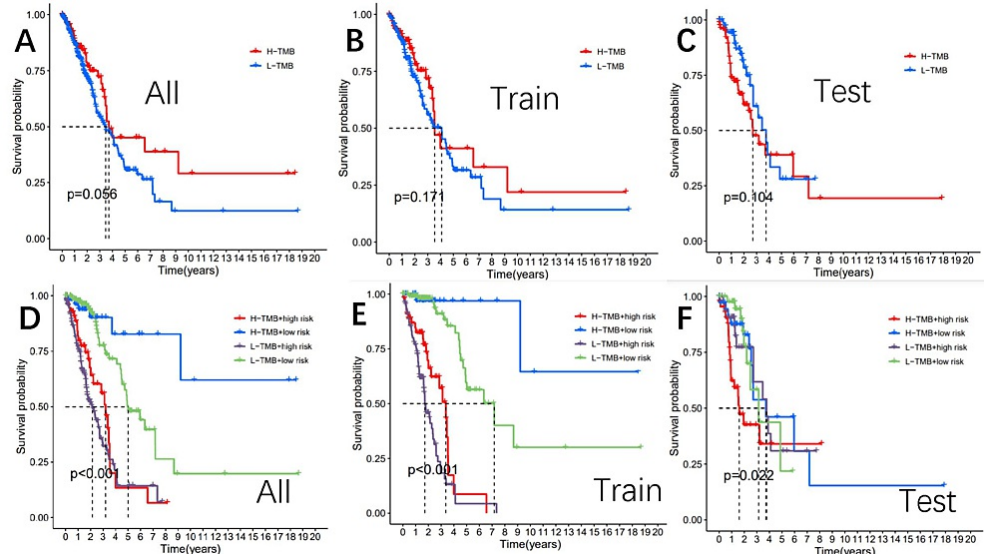
The survival curve revealed that survival time varies among TMB-risk scores. The entire cohort (A), the training cohort (B), and the testing cohort (C) The survival of the TMB+ risk subgroup in the entire cohort (D), train cohort (E), and test cohort (F) TMB: tumor mutation burden

Studies have shown that higher TMB is associated with overall survival after immunotherapy for various cancer types, suggesting that TMB can be used as a predictive biomarker for the therapeutic efficacy of immune checkpoint inhibitors [27]. We compared TMB between the high and low-risk subgroups and found no significant difference in the overall cohort (p=0.066), the test cohort (p=0.31), or the training cohort (p=0.11) (p=0.05). As expected, the TMB survival curves for the entire cohort (P =0.056), the test cohort (p=0.104), and the training cohort (p=0.171) were not statistically significant (Figures 6A-6C).

We visually demonstrated the relationship between TMB, high and low-risk subgroups, and survival rates by developing survival curves, which were divided into four groups: H-TMB+ high-risk subgroup, H-TMB+ low-risk subgroup, L-TMB+ high-risk subgroup, and L-TMB+ low-risk subgroup. In the entire cohort, a statistically significant difference was observed between the four groups (P <0.001), with the highest survival probability observed in the H-TMB+ low-risk subgroup, followed by the L-TMB+ low-risk subgroup, and finally the H-TMB+ high-risk subgroup. The training and test groups are illustrated in Figure 6D-F.

Analysis of tumor immune escape and immunotherapy

**Figure 7:**
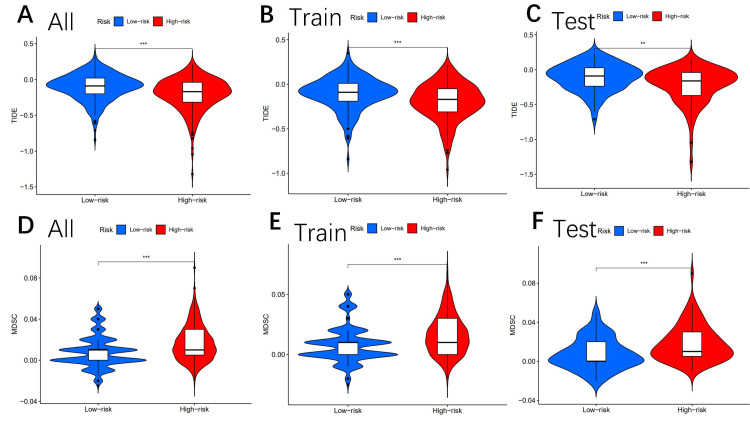
Tumor microenvironment results based on genes. Analysis of the differences among TIDE (A–C) and MDSC (D–F) in different risk subgroups "*" means that p-value<0.05, "**" means that p-value <0.01, "***" means that p-value<0.001, and "ns" means no significance TIDE: tumor immune dysfunction and rejection; MDSC: myeloid-derived suppressor cells

We also analyzed tumor immune escape and immunotherapy for related lncRNAs in both high- and low-risk subgroups. Surprisingly, the TIDE scores in either group were lower in the high-risk subgroup, higher in the low-risk subgroup, and statistically significant in the low-risk subgroup (p<0.01, **), as shown in Figures 7A-7C.

**Figure 8:**
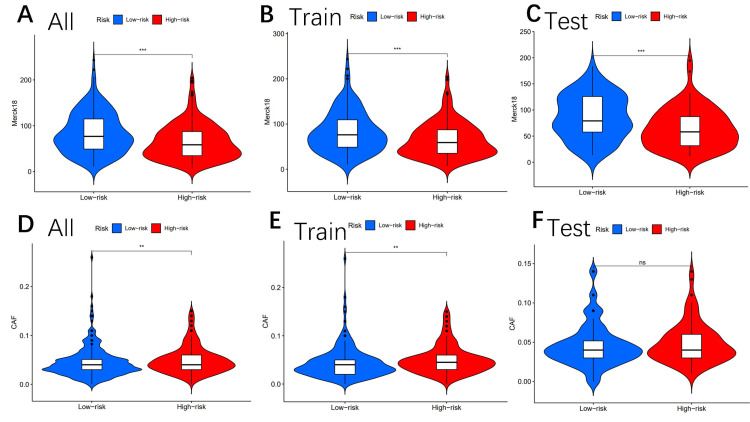
Tumor microenvironment results based on genes Analysis of the differences among MERCK18 (A–C) and CAF (D–F) in different risk subgroups "*" means that p-value<0.05, "**" means that p-value <0.01, "***" means that p-value<0.001, and "ns" means no significance CAF: cancer-associated fibroblast

The differences in MDSC between the high-risk and low-risk groups were also statistically significant (Figures 7D-7F). Moreover, MERCK18 was statistically significant between the high- and low-risk subgroups (Figures 8A-8C).

**Figure 9:**
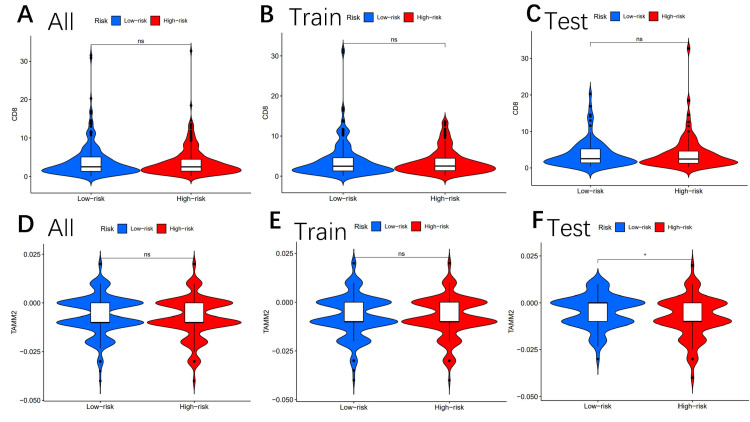
Tumor microenvironment results based on genes Analysis of the differences among CD8 (A-C) and TAAM2 (D-F) in different risk subgroups "*" means that p-value<0.05, "**" means that p-value <0.01, "***" means that p-value<0.001, and "ns" means no significance

**Figure 10:**
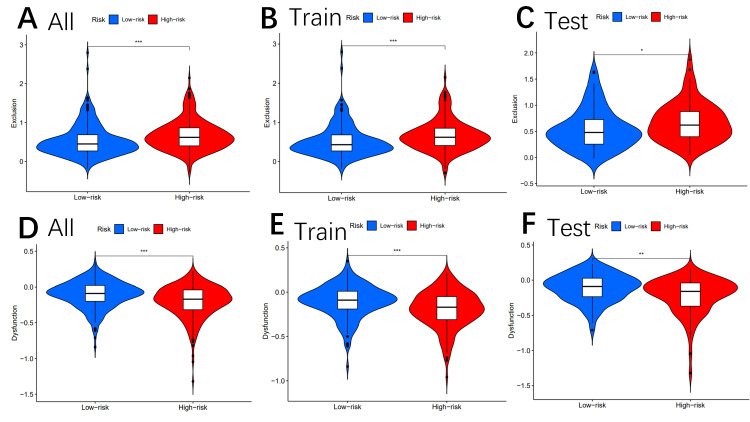
Tumor microenvironment results based on genes Analysis of the differences among exclusion (A–C) and dysfunction (D–F) in different risk subgroups "*" means that p-value<0.05, "**" means that p-value <0.01, "***" means that p-value<0.001, and "ns" means no significance.

**Figure 11:**
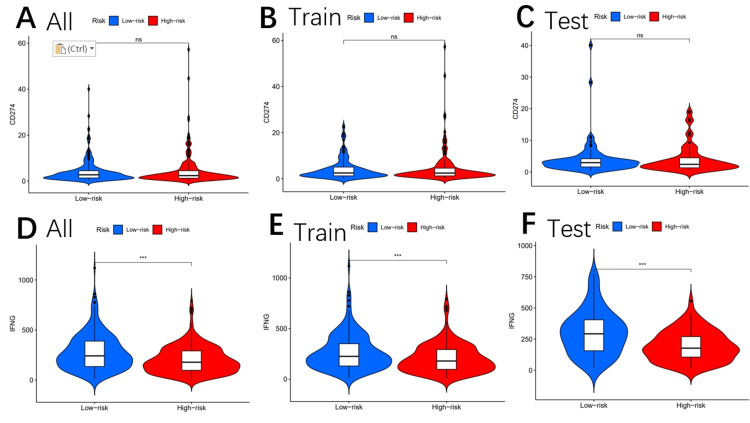
Tumor microenvironment results based on genes Analysis of the differences among CD274 (A–C) and IFNG (D–F) in different risk subgroups "*" means that p-value<0.05, "**" means that p-value <0.01, "***" means that p-value<0.001, and "ns" means no significance. INFG: interferon-gamma

Other immune markers, including CAF (Figures 8D-8F), CD8 (Figures 9A-9C), TAAM2 (Figures 9D-9F), exclusion (Figures 10A-10C), dysfunction (Figures 10D-10F), CD274 (Figures 11A-11C), and IFNG (Figures 11D-11F), were also analyzed.

Screening of tumor chemotherapy drugs

**Figure 12:**
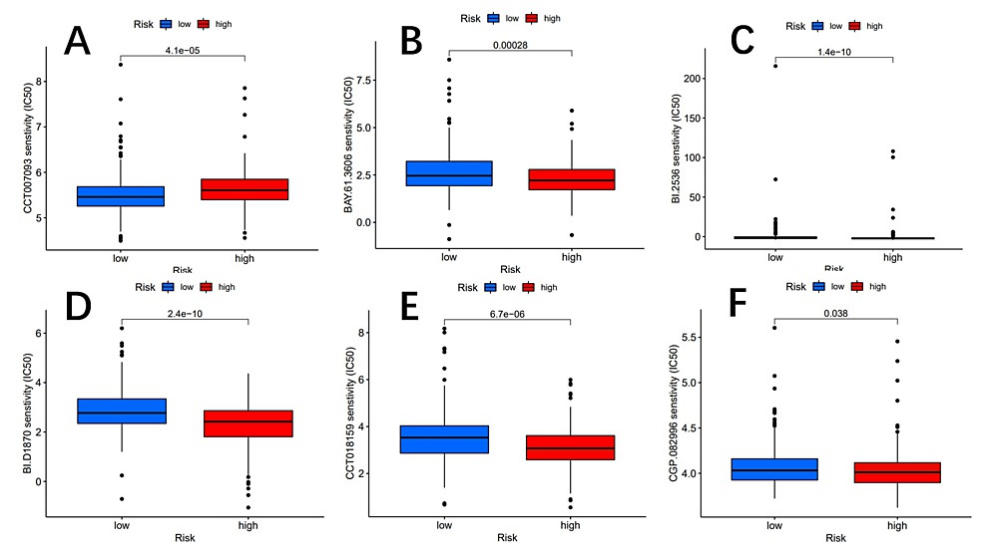
The chart shows six potential drugs for NSCLC. Potential drugs include CCT007093 (A), BAY.61.3606 (B), BI.2536 (C), BI.D1870 (D), CCT018159 (E), and CGP.082996 (F). NSCLC: non-small cell lung cancer

**Figure 13:**
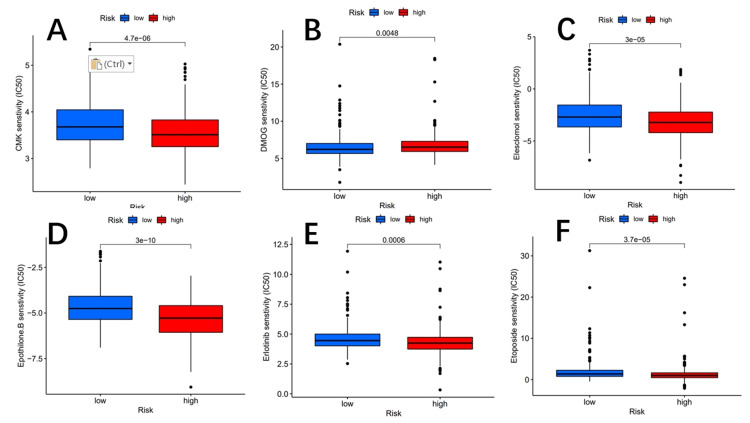
The chart shows six potential drugs for NSCLC. Potential drugs include CMK (A), DMOG (B), elesclomol (C), epothilone B (D), erlotinib (E), and etoposide (F). NSCLC: non-small cell lung cancer; DMOG: dimethyloxalylglycine

To accurately identify potential drugs targeted to our lncRNA model therapy, we used the pRRophetic algorithm based on the half-maximum inhibitory concentration (IC50) available in the Genomics of Drug Sensitivity in Cancer (GDSC) database [28]. The algorithm screened for 60 compounds, all of which were statistically significant in the high-risk and low-risk subgroups, with significant differences in estimated semi-inhibitory concentrations between the two groups. The high-risk subgroup was more sensitive to most compounds, whereas PPM1D was more sensitive to low-risk drugs. The lower panel shows 15 compounds that may be used in lung cancer patients, as illustrated in Figures 12-13.

**Figure 14:**
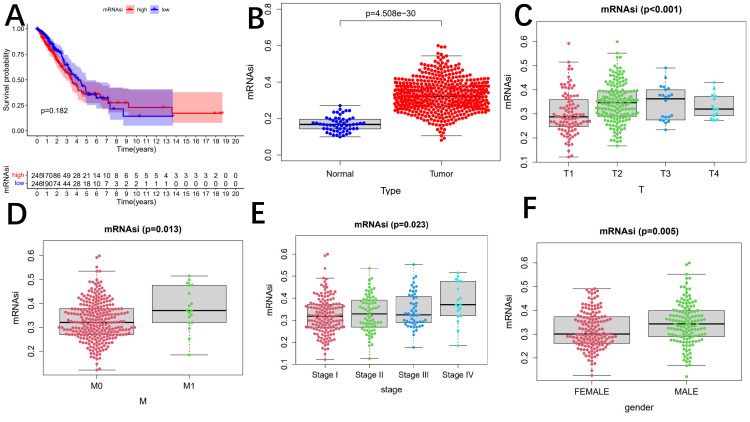
The mRNA stability index (mRNAsi) analysis There is no significant difference in overall survival among different risk groups (A). The mRNA stability index (mRNAsi) exhibited a notable distinction between normal and tumor tissues (B). Significant variations in mRNAsi were observed among clinicopathological factors (C–F).

The mRNA expression-based stemness (mRNAsi) index is an index calculated based on gene expression data. Unfortunately, we found that mRNA and OS were not strongly correlated between the high-risk and low-risk groups (P =0.182) (Figure 14A).

Next, we compared the stem cell index of normal and tumor tissues and found a significant correlation between the two (Figure 14B). We also analyzed the correlations between mRNAsi and clinical data, including primary tumor, distant metastasis, stage, and sex (Figure 14C-F). The stem cell index of patients with stage M1 and M0 lung cancer was statistically significant (P =0.013). Additionally, the mRNAsi of men was higher than that of women.

## Discussion

Single-cell RNA sequencing technology has been utilized to advance the study of tumor microenvironments in various cancer types, such as breast cancer [29], pancreatic ductal adenocarcinoma [30], and NSCLC [31]. A more profound comprehension of immune-tumor interactions during lung cancer through single-cell sequencing technology can aid in identifying novel immunotherapeutic targets [32]. Recent studies have shown that immunotherapy is a highly promising therapy in addition to traditional surgery, radiotherapy, chemotherapy, and molecular targeted therapy [33, 34]. For instance, the fragile histidine triad (FHIT) provides a new therapeutic target and strategy for the clinical treatment of lung cancer, which further enhances the therapeutic effect of lung cancer [35]. The gene FNBP1 can even impact the prognosis of cancer. High expression of FNBP1 has a favorable prognosis for breast cancer and lung adenocarcinoma but an unfavorable prognosis for gastric adenocarcinoma [36]. Cancer remains a significant cause of human mortality, and lung cancer plays a crucial role in it. The absence of early diagnostic methods for LUAD results in a poor prognosis, and the five-year survival rate of patients is usually less than 15% [37, 38]. In conclusion, a more profound comprehension of the relationship between LUAD and immunotherapy is of paramount importance in discovering more precise diagnostic and prognostic biomarkers. In this study, we employed the method of lncRNAs associated with marker genes found by single-cell sequencing to analyze the prognostic characteristics, TME, and drug resistance analysis of lncRNAs and NSCLC patients.

In this study, we have demonstrated the significance of related lncRNAs in patients with NSCLC. Firstly, we identified 28 differentially expressed genes in the training group, including AC105290.1, AC108529.1, AC022165.1, AL139099.3, FAM66C, PAN3-AS, C5orf66, AC025741.1, AC010999.2, ZNF571-AS1, IER3-AS1, AC026310.2, AC015967.1, AC080023.1, AC105036.3, AC025419.1, AC092809.3, LINC01150, LINC02410, and AP003500.1, AL031600.2, LINC01776, LINC02448, ID2-AS1, AC012409.4, MAP3K20-AS1, AC006460.1, and AL157931.1. These lncRNAs possess prognostic risk characteristics, which we utilized in our survival analysis. The results indicated that patients with NSCLC in the high-risk subgroup had significantly lower overall survival than those in the low-risk subgroup. AC025419.1 and LINC02448 showed significant expression, with low expression in the high-risk group and high expression in the low-risk group. These results were consistent in both the test and whole groups. Analysis of the relationship between risk scores and clinical factors revealed that early tumors were associated with the low-risk subgroup, while advanced tumors were significantly associated with the high-risk subgroup. Univariate regression analysis showed that AJCC stage, T stage, N stage, and risk score were significantly associated with OS, while multivariate regression analysis showed that only risk score was significantly associated with OS. To predict the one-year, three-year, and five-year survival rates of patients, we used the ROC curve, which can simultaneously compare the predictive effects of risk characteristics and clinicopathological factors [39]. The C-index curve verified that the risk characteristics of the ROC curve were better than the results of clinicopathological factors. We chose the nomogram, a visual model that can be compared with the ideal model, and the calibration maps of one-year, five-year, and 10-year OS rates can be well predicted. The GO/KEGG analysis gave us a hint that gene sets have a potential relationship with biological metabolism. In addition, drug analysis showed that PPM1D may have some efficacy. The gene PPM1D may be associated with tumor immune cell infiltration in hepatocellular carcinoma (HCC), thus indicating that PPM1D may be a potential prognostic biomarker for cancer progression [40]. Furthermore, studies have confirmed that PPM1D is the target gene of mir-16 in the A459 lung cancer cell line. However, potential inhibitors of PPM1D are scarce due to the unclear structure of PPM1D [41]. While PPM1D deficiency is well-tolerated in mice, emerging evidence suggests that PPM1D deficiency in the immune system induces an inflammatory environment [42].

The complexity and diversity of the immune microenvironment influence the responsiveness of immunotherapy. The low-risk pathways for promoting various immune microenvironment characteristics are HLA, cytolytic activity, and inflammation-promoting, as indicated by the risk model. Lin developed and validated a hypoxia risk model that can serve as an independent prognostic indicator, reflecting the strength of the overall immune response in the glioma microenvironment [43]. In addition to the relationship between the risk model and the immune microenvironment, we also demonstrated that the mutational burden analysis did not yield statistically significant results between the high-risk and low-risk groups in TMB, but it was statistically significant between the high-risk and low-risk groups in the TIDE score. The TIDE scores of MDSC and MERCK18 were significantly correlated with the risk scores. MDSC is a major inhibitor of T lymphocyte activation [44]. Therefore, more and more data prove that MDSC is an effective target for immune resistance and utilization of the immune checkpoint blockade (ICB) effect [45]. Subsequently, we attempted various anticancer treatments and identified some that may be relevant to the treatment of non-small cell lung cancer, such as PPM1D.

We engaged in a comprehensive analysis of tumor stem cell indices, revealing a potential correlation between tumor stem cells and the immune microenvironment. The intricate involvement of tumor stem cells in tumor treatment was unveiled. These cells play a crucial role in promoting abnormalities in apoptosis, thus fostering therapy resistance [46]. Interestingly, our study demonstrated that the stem cell index did not exhibit a significant correlation with OS in either the high-risk or low-risk groups. This finding suggests that tumor progression is not directly associated with OS. However, certain pathways were found to be linked to tumor progression. For instance, fructose-1,6-bisphosphatase (FBP1) inhibits tumor progression in LUAD, while hypoxia-mediated glycogen amylase (GBE1) can promote tumor progression [47]. Notably, the hypoxia risk score displayed a positive correlation with the enrichment score of most immunotherapy-positive gene features [48]. The substantial disparities observed in clinicopathological factors and mRNAsi expression further validated the reliability of nomograms in predicting patient prognosis.

Like all studies, our research also possesses certain limitations. This study mainly relied on data from public databases, which may have resulted in incomplete data and limited clinical information. This limits our comprehensive understanding of patient medical history, treatment history, and other key clinical parameters. Although we have identified lncRNAs associated with NSCLC, these results still require further experimental validation to determine their exact functions in tumor development and treatment. Before experimental verification, the reliability of the conclusions may be affected. Some research conclusions may appear too speculative and require more experimental evidence and data support to strengthen their scientific credibility. The data cutoff for the study is 2022, and due to the rapid development in the field of lung cancer research and treatment, some of the latest advances and treatments may not have been considered in this study. Finally, although our study discovered lncRNAs with potential clinical value, these results require more clinical studies to verify their effectiveness and application in actual patients.

## Conclusions

In summary, we successfully identified 28 metabolically associated lncRNAs based on prognostic models for NSCLC patients, enabling risk characteristics to predict OS. Subsequent GO/KEGG pathway enrichment analysis revealed the involvement of lncRNAs in nitrogen metabolism. Furthermore, we investigated HLA, cytolytic activity, and inflammation-promoting pathways as protective factors within the immune microenvironment. The results of TMB were found to be associated with survival in NSCLC patients. Notably, MDSC, MERCK18, and risk score exhibited a significant positive correlation. Survival rates exhibited significant differences between high-risk and low-risk groups, although cancer development did not show an association with the risk score subgroup. Drug therapy remains a viable treatment option, thus making drug screening an indispensable component.

## References

[1] Wen L, Tang F (2018). Boosting the power of single-cell analysis. Nat Biotechnol.

[2] Tang F, Barbacioru C, Wang Y (2009). mRNA-seq whole-transcriptome analysis of a single cell. Nat Methods.

[3] Ni X, Zhuo M, Su Z (2013). Reproducible copy number variation patterns among single circulating tumor cells of lung cancer patients. Proc Natl Acad Sci U S A.

[4] Lipinski KA, Barber LJ, Davies MN, Ashenden M, Sottoriva A, Gerlinger M (2016). Cancer evolution and the limits of predictability in precision cancer medicine. Trends Cancer.

[5] Jiwnani S, Penumadu P, Ashok A, Pramesh CS (2022). Lung cancer management in low and middle-income countries. Thorac Surg Clin.

[6] He J, Li N, Chen WQ (2021). China guideline for the screening and early detection of lung cancer(2021, Beijing) (Article in Chinese). Zhonghua Zhong Liu Za Zhi.

[7] Torre LA, Siegel RL, Jemal A (2016). Lung cancer statistics. Adv Exp Med Biol.

[8] Marusyk A, Polyak K (2010). Tumor heterogeneity: causes and consequences. Biochim Biophys Acta.

[9] Kim KT, Lee HW, Lee HO (2016). Application of single-cell RNA sequencing in optimizing a combinatorial therapeutic strategy in metastatic renal cell carcinoma. Genome Biol.

[10] Eberwine J, Sul JY, Bartfai T, Kim J (2014). The promise of single-cell sequencing. Nat Methods.

[11] Normanno N, Rachiglio AM, Roma C (2013). Molecular diagnostics and personalized medicine in oncology: challenges and opportunities. J Cell Biochem.

[12] Epp CD (1997). Definition of a gene. Nature.

[13] Franzén O, Gan LM, Björkegren JL (2019). PanglaoDB: a web server for exploration of mouse and human single-cell RNA sequencing data. Database (Oxford).

[14] Tomczak K, Czerwińska P, Wiznerowicz M (2015). The Cancer Genome Atlas (TCGA): an immeasurable source of knowledge. Contemp Oncol (Pozn).

[15] Zhang Z, Li H, Jiang S, Li R, Li W, Chen H, Bo X (2019). A survey and evaluation of Web-based tools/databases for variant analysis of TCGA data. Brief Bioinform.

[16] Sachs MC (2017). plotROC: a tool for plotting ROC curves. J Stat Softw.

[17] Hänzelmann S, Castelo R, Guinney J (2013). GSVA: gene set variation analysis for microarray and RNA-seq data. BMC Bioinformatics.

[18] Addeo A, Friedlaender A, Banna GL, Weiss GJ (2021). TMB or not TMB as a biomarker: that is the question. Crit Rev Oncol Hematol.

[19] Palmeri M, Mehnert J, Silk AW (2022). Real-world application of tumor mutational burden-high (TMB-high) and microsatellite instability (MSI) confirms their utility as immunotherapy biomarkers. ESMO Open.

[20] Liu Z, Wang L, Liu L, Lu T, Jiao D, Sun Y, Han X (2021). The identification and validation of two heterogenous subtypes and a risk signature based on ferroptosis in hepatocellular carcinoma. Front Oncol.

[21] Fu J, Li K, Zhang W, Wan C, Zhang J, Jiang P, Liu XS (2020). Large-scale public data reuse to model immunotherapy response and resistance. Genome Med.

[22] Feng G, Xue F, He Y, Wang T, Yuan H (2021). The identification of stemness-related genes in the risk of head and neck squamous cell carcinoma. Front Oncol.

[23] Zhang Y, Tseng JT, Lien IC, Li F, Wu W, Li H (2020). mRNAsi index: machine learning in mining lung adenocarcinoma stem cell biomarkers. Genes (Basel).

[24] Malta TM, Sokolov A, Gentles AJ (2018). Machine learning identifies stemness features associated with oncogenic dedifferentiation. Cell.

[25] Li N, Li Y, Zheng P, Zhan X (2021). Cancer stemness-based prognostic immune-related gene signatures in lung adenocarcinoma and lung squamous cell carcinoma. Front Endocrinol (Lausanne).

[26] Justiz Vaillant AA, Qurie A (2023). Immunodeficiency. StatPearls. Treasure Island.

[27] Chan TA, Yarchoan M, Jaffee E, Swanton C, Quezada SA, Stenzinger A, Peters S (2019). Development of tumor mutation burden as an immunotherapy biomarker: utility for the oncology clinic. Ann Oncol.

[28] Che Y, Jiang D, Xu L (2022). The clinical prediction value of the ubiquitination model reflecting the immune traits in LUAD. Front Immunol.

[29] Azizi E, Carr AJ, Plitas G (2018). Single-cell map of diverse immune phenotypes in the breast tumor microenvironment. Cell.

[30] Peng J, Sun BF, Chen CY (2019). Single-cell RNA-seq highlights intra-tumoral heterogeneity and malignant progression in pancreatic ductal adenocarcinoma. Cell Res.

[31] Lambrechts D, Wauters E, Boeckx B (2018). Phenotype molding of stromal cells in the lung tumor microenvironment. Nat Med.

[32] Beaumont KG, Beaumont MA, Sebra R (2020). Application of single-cell sequencing to immunotherapy. Urol Clin North Am.

[33] Somasundaram A, Burns TF (2017). The next generation of immunotherapy: keeping lung cancer in check. J Hematol Oncol.

[34] Yang L, Wang L, Zhang Y (2016). Immunotherapy for lung cancer: advances and prospects. Am J Clin Exp Immunol.

[35] Situ Y, Gao R, Lei L, Deng L, Xu Q, Shao Z (2022). System analysis of FHIT in LUAD and LUSC: the expression, prognosis, gene regulation network, and regulation targets. Int J Biol Markers.

[36] Wang Z, Tian Z, Song X, Zhang J (2022). Membrane tension sensing molecule-FNBP1 is a prognostic biomarker related to immune infiltration in BRCA, LUAD and STAD. BMC Immunol.

[37] Spella M, Stathopoulos GT (2021). Immune resistance in lung adenocarcinoma. Cancers (Basel).

[38] Šutić M, Vukić A, Baranašić J (2021). Diagnostic, predictive, and prognostic biomarkers in non-small cell lung cancer (NSCLC) management. J Pers Med.

[39] Albeck MJ, Børgesen SE (1990). ROC-curve analysis. A statistical method for the evaluation of diagnostic tests (Article in Danish). Ugeskr Laeger.

[40] Yu Z, Song Y, Cai M (2021). PPM1D is a potential prognostic biomarker and correlates with immune cell infiltration in hepatocellular carcinoma. Aging (Albany NY).

[41] Deng W, Li J, Dorrah K (2020). The role of PPM1D in cancer and advances in studies of its inhibitors. Biomed Pharmacother.

[42] Uyanik B, Grigorash BB, Goloudina AR, Demidov ON (2017). DNA damage-induced phosphatase Wip1 in regulation of hematopoiesis, immune system and inflammation. Cell Death Discov.

[43] Lin W, Wu S, Chen X (2020). Characterization of hypoxia signature to evaluate the tumor immune microenvironment and predict prognosis in glioma groups. Front Oncol.

[44] Li T, Liu T, Zhu W (2021). Targeting MDSC for immune-checkpoint blockade in cancer immunotherapy: current progress and new prospects. Clin Med Insights Oncol.

[45] Oliva M, Spreafico A, Taberna M, Alemany L, Coburn B, Mesia R, Siu LL (2019). Immune biomarkers of response to immune-checkpoint inhibitors in head and neck squamous cell carcinoma. Ann Oncol.

[46] Dawood S, Austin L, Cristofanilli M (2014). Cancer stem cells: implications for cancer therapy. Oncology (Williston Park).

[47] Li L, Yang L, Fan Z (2020). Hypoxia-induced GBE1 expression promotes tumor progression through metabolic reprogramming in lung adenocarcinoma. Signal Transduct Target Ther.

[48] Liu Z, Tang Q, Qi T (2021). A robust hypoxia risk score predicts the clinical outcomes and tumor microenvironment immune characters in bladder cancer. Front Immunol.

